# INFECTION PROFILE IN HIP ARTHROPLASTY AT A REFERRAL HOSPITAL

**DOI:** 10.1590/1413-785220263403e299587

**Published:** 2026-06-12

**Authors:** Flávia Rachel Nogueira de Negreiros Freitas, Danilo do Nascimento Viana, Isânio Vasconcelos Mesquita

**Affiliations:** 1 Universidade Estadual do Piaui, Departamento de Ortopedia, Teresina, PI, Brazil.

**Keywords:** Arthroplasty, Epidemiology, Cross Infection, Prostheses and Implants, Artroplastia, Epidemiologia, Infecção hospitalar, Próteses e implantes

## Abstract

**Objective:**

To analyze the occurrence of infections in patients undergoing hip arthroplasty at a tertiary hospital in Teresina, Piauí, Brazil, between 2020 and 2023.

**Methods:**

A retrospective, longitudinal, and observational study conducted at Hospital Getúlio Vargas, Teresina, Piauí, with patients who developed prosthesis-related infections during the study period.

**Results:**

A total of 1,420 surgeries were performed, with an infection rate of 2.0% (22 cases). Female patients predominated (59.1%) with a mean age of 68.8 years. Trauma was the main surgical indication (77.3%). Total arthroplasty was performed in 68.2% of cases, with 59.1% done without cement. The most common pathogens were Escherichia coli (22.7%) and Acinetobacter baumannii (18.2%). The main antibiotics used were ciprofloxacin (31.7%), clindamycin (25%), and vancomycin (15%). Hospital discharge occurred in 86.4% of cases, with no significant association between variables and outcomes (p > 0.05).

**Conclusion:**

The findings highlight the importance of preventive protocols and surveillance to reduce infections in hip arthroplasty. ***Level of Evidence II; Retrospective Study.***

## RESUMO

**Objetivo:**

Analisar a ocorrência de infecções em pacientes submetidos à artroplastia de quadril no Hospital Getúlio Vargas, Teresina, Piauí, entre 2020 e 2023.

**Método:**

Estudo retrospectivo, longitudinal e observacional realizado no Hospital Getúlio Vargas, com pacientes que apresentaram infecção relacionada à prótese no período.

**Resultados:**

Foram realizadas 1.420 cirurgias, com taxa de infecção de 2,0% (22 casos). Predominou o sexo feminino (59,1%) e a média de idade foi de 68,8 anos. Trauma foi a principal indicação (77,3%). Artroplastia total foi realizada em 68,2%, e 59,1% sem uso de cimento. Os patógenos mais comuns foram Escherichia coli (22,7%) e Acinetobacter baumannii (18,2%). Os principais antibióticos usados foram Ciprofloxacino (31,7%), Clindamicina (25%) e Vancomicina (15%). A alta hospitalar ocorreu em 86,4% dos casos, sem associação significativa entre as variáveis e os desfechos (p > 0,05).

**Conclusão:**

Destaca-se a importância de protocolos preventivos e de vigilância para reduzir infecções em artroplastias de quadril. ***Nível de Evidência II; Estudo Retrospectivo.***

## INTRODUCTION

Hip arthroplasty has become a widely used surgical approach to restore mobility and improve the quality of life for patients suffering from pain and limitations caused by conditions such as osteoarthritis or inflammatory arthritis^1^. Despite the significant functional benefits, the procedure still requires special care in the postoperative period, especially regarding the risk of infections. These complications, although infrequent, can compromise the expected outcomes and require more complex and prolonged therapeutic strategies than in cases without infection, making careful follow-up of these patients essential^1,2^.

Surgical Site Infections (SSI) are one of the main complications in the postoperative period, being associated with increased morbidity, prolonged hospitalization, and elevated healthcare costs^3^. Various risk factors contribute to the occurrence of these infections, including advanced age, frailty, presence of comorbidities such as diabetes mellitus, as well as the complexity and duration of the surgical procedure^4^.

In the realm of orthopedic surgeries, periprosthetic joint infection (PJI) stands out as a complication that occurs after the implantation of joint prostheses, such as hip prostheses^3^. Considered one of the most serious complications following total hip arthroplasty, PJI has an increased risk in the presence of comorbidities^5^. In addition to significantly compromising the patient's health, potentially leaving them in worse condition than before the surgery^6^, this infection frequently results in hospital readmissions and, in many cases, requires revision surgical procedures, contributing to a substantial increase in hospital costs and the associated economic impact^7^.

According to clinical and laboratory criteria established by international guidelines, the diagnosis of PJI is confirmed by findings such as positive deep tissue cultures, fistula with joint communication, or the presence of inflammatory markers and changes in synovial fluid. Considering its severity and impact on the clinical evolution of patients, monitoring the incidence of SSI, especially in arthroplasties, is essential for improving care protocols and prevention strategies^3^. Since 2013, an international consensus has established specific diagnostic criteria for periprosthetic infection, underscoring the importance of detailed clinical data, including microbiological cultures, histological findings, and synovial fluid examinations. However, the unavailability of this data in national information systems, such as DATASUS, limits the application of these criteria in population studies in Brazil, highlighting the need for improvements in the standardization and quality of health records^8^.

The treatment of periprosthetic infection varies according to the severity, the time of onset of the infection, and the clinical conditions of the patient^9^. Therapeutic options may include anything from the administration of antibiotics with preservation of the implant to more invasive procedures, such as prosthesis replacement in one or two surgical stages or, in extreme cases, resection arthroplasty (Girdlestone procedure)^10^.

The choice of treatment should be individualized, taking into account factors such as prosthesis stability, presence of fistula, systemic inflammatory response, and microbiological profile of the infection, with the aim of eradicating the infectious agent, preserving joint function, and reducing recurrence and associated morbidity rates^11^. In this context, hip prosthesis infection is one of the greatest challenges in the postoperative period of arthroplasty, both for patients and healthcare professionals. In addition to potentially significantly prolonging the length of hospitalization, these infections often require surgical reinterventions and can lead to severe conditions, including death. Factors such as advanced age, associated comorbidities, and previous clinical status increase patients’ susceptibility to these complications. In this scenario, investigating the incidence of infection in hip arthroplasties becomes essential to strengthen prevention strategies, early diagnosis, and appropriate treatment, promoting better clinical outcomes and contributing to the sustainability of healthcare systems.

## OBJECTIVES

The overall objective of this study is to analyze the incidence of infections associated with hip prostheses in patients undergoing arthroplasty procedures at a tertiary hospital in Teresina, Piauí. Specifically, the aim is to calculate the incidence rate of surgical site infection (SSI) in patients undergoing hip arthroplasty at Getúlio Vargas Hospital from 2020 to 2023; analyze the association between the occurrence of SSI and clinical and surgical factors, such as type of anesthesia, type of arthroplasty (partial or total), ASA classification, age, need for ICU admission, use of antibiotic prophylaxis, use of cement, history of previous orthopedic surgery, and cause of fracture; verify the types of antimicrobials administered in the treatment of surgical site infections; and identify the microorganisms involved in these infections.

## METHODOLOGY

This is a mixed, longitudinal, retrospective observational study with a descriptive approach, conducted at Getúlio Vargas Hospital (GVH). The sample was selected for convenience, including patients undergoing arthroplasty procedures who presented prosthesis-related infections from 2020 to 2023.

Patients who underwent hip prosthesis insertion through arthroplasty surgery at GVH during this period were included, while those without a diagnosis of local or systemic infection related to the prosthesis, as well as those with incomplete, illegible, or inconsistent medical records, were excluded. In the end, the analysis included 22 participants.

Data collection was performed through a systematic analysis of the medical records of the selected patients, using a standardized instrument to ensure uniformity and reliability. General information was extracted, such as age, sex, and comorbidities; data related to hospitalization, including admission date, length of hospital stay, and procedures performed; clinical information related to the infection, such as signs, symptoms, and laboratory and microbiological results; as well as data on clinical evolution, instituted treatments, and outcomes.

The collected data were organized and tabulated in Microsoft Excel 2016 and subsequently analyzed using SPSS version 26.0. Initially, descriptive analysis was performed, presenting qualitative variables by absolute and relative frequencies, and quantitative variables by measures of central tendency (mean and median) and dispersion (standard deviation). To verify associations between variables, the Pearson chi-square test was used, with Yates’ correction for continuity when necessary. The significance level adopted was 5% (p < 0.05), with a 95% confidence interval.

The study strictly followed the ethical principles established by Resolution No. 466/2012 of the National Health Council, having been approved by the Research Ethics Committee with Human Beings of the institution under opinion No. 6.835.596 and CAAE 77819923.7.0000.5209, ensuring confidentiality, anonymity, and responsible use of information.

## RESULTS

**Figure 1:**
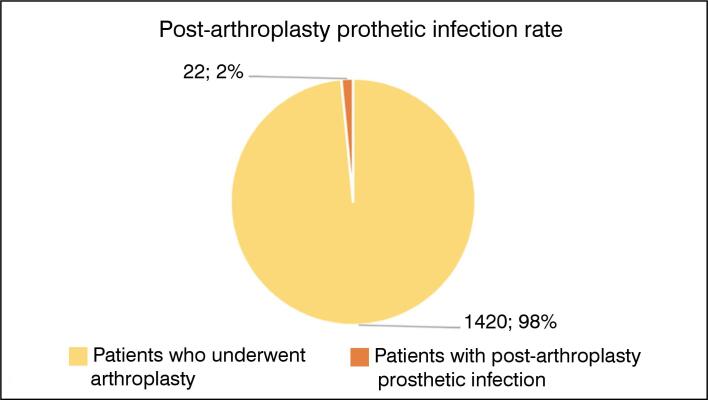
Occurrence of surgical site infection among patients undergoing arthroplasty at Getúlio Vargas Hospital from 2020 to 2023, Teresina-PI. (n = 22).

Between 2020 and 2023, 1,420 hip arthroplasties were performed at Getúlio Vargas Hospital in the city of Teresina, PI. Among these patients, a surgical site infection rate of 2.0% was observed, totaling 22 cases. (Figure 1)

Table 1 presents the social and clinical characteristics of surgical site infections in patients undergoing hip arthroplasty at Getúlio Vargas Hospital – PI, from 2020 to 2023.

**Table 1:** Social and clinical characteristics of surgical site infections resulting from hip arthroplasties, based on information from medical records of patients undergoing hip arthroplasty at Getúlio Vargas Hospital – PI, from 2020 to 2023. (n = 22).

Variables	N(%)	IC-95%	Mean (CI-95)	SD
**Social Profile**				
**Gender**				
Male	9(40.9)	(22.5-61.5)		
Female	13(59.1)	(38.5-77.5)		
Age Range			68.82(62.75-74.89)	13.69
40-59 Years	5(22.7)	(9.2-42.9)		
≥60 Years	17(77.3)	(57.1-90.8)		
**Clinical Profile**				
**Cause**				
Hip Osteoarthritis	4(18.2)	(6.5-37.6)		
Avascular Necrosis	1(4.5)	(0.5-19.3)		
Trauma	17(77.3)	(57.1-90.8)		
**Anesthesia**				
Spinal	22(100.0)	(62.4-93.5)		
**ASA**				
1	4(18.2)	(6.5-37.6)		
2	10(45.5)	(26.3-65.7)		
3	8(36.4)	(18.9-57.1)		
**UTI**				
No	10(45.5)	(26.3-65.7)		
Yes	12(54.5)	(34.3-73.7)		
**Prophylactic Antibiotic**				
Cephalothin 48h	3(13.6)	(4.0-32.1)		
Cephalothin 24h	19(86.4)	(67.9-96.0)		
**Previous Surgery**				
No	20(90.9)	(73.9-98.1)		
Yes	2(9.1)	(1.9-26.1)		
**Previous Surgery (type)**				
Dislocation revision	1(50.0)	(6.1-93.9)		
Trochanteric	1(50.0)	(6.1-93.9)		
**Partial or total**				
Partial	7(31.8)	(15.5-52.6)		
Total	15(68.2)	(47.4-84.5)		
**Cement**				
With cement	9(40.9)	(22.5-61.5)		
Without cement	13(59.1)	(38.5-77.5)		

Source: Authors (2025). ^1^CI-95% for proportion, at the 5% level. ^2^CI-95% for mean, at the 5% level. Med: Median/ SD: Standard Deviation.

Regarding the sociodemographic profile, a predominance of female sex (59.1%) was observed, with an average age of 68.8 years and a higher frequency of patients aged 60 years or older (77.3%), indicating greater vulnerability among the elderly to postoperative complications.

Clinically, the main surgical indication was trauma (77.3%), followed by hip osteoarthritis (18.2%) and avascular necrosis (4.5%). All patients underwent spinal anesthesia, suggesting a standardized anesthetic protocol. The majority were classified as ASA class 2 (45.5%) and 3 (36.4%), reflecting prior clinical impairment.

More than half (54.5%) required ICU admission, highlighting the severity of the cases. Antibiotic prophylaxis with cephalothin for 24 hours was adopted in 86.4% of patients. Regarding surgical technique, total arthroplasty predominated (68.2%), with cementless fixation (59.1%).

Figure 1 illustrates the distribution of bacteria identified in surgical site infections. The most prevalent pathogens were *Escherichia coli* (22.7%) and *Acinetobacter baumannii* (18.2%), followed by *Staphylococcus aureus*, *Pseudomonas aeruginosa*, and other bacteria in smaller proportions. The predominance of *E. coli* may indicate contamination of endogenous origin, while *S. aureus* is a common pathogen in hospital and surgical infections.

**Figure 2:**
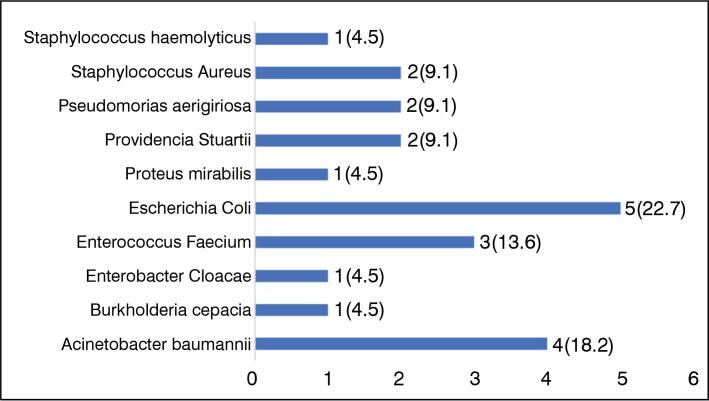
Characteristics of surgical site bacteria resulting from hip arthroplasties, based on information from medical records of patients who underwent hip arthroplasty at Getúlio Vargas Hospital – PI, from 2020 to 2023. (n = 22).

Figure 2 shows the bacteria isolated in surgical site infections after hip arthroplasty. *Escherichia coli* (22.7%) and *Acinetobacter baumannii* (18.2%) were the most prevalent, followed by *Enterococcus faecium* (13.6%). Other pathogens, such as *Staphylococcus aureus*, *Pseudomonas aeruginosa*, and *Providencia stuartii*, appeared with lower frequency. The presence of *E. coli* may indicate endogenous contamination, while *A. baumannii* and *S. aureus* reflect common hospital infections.

**Figure 3:**
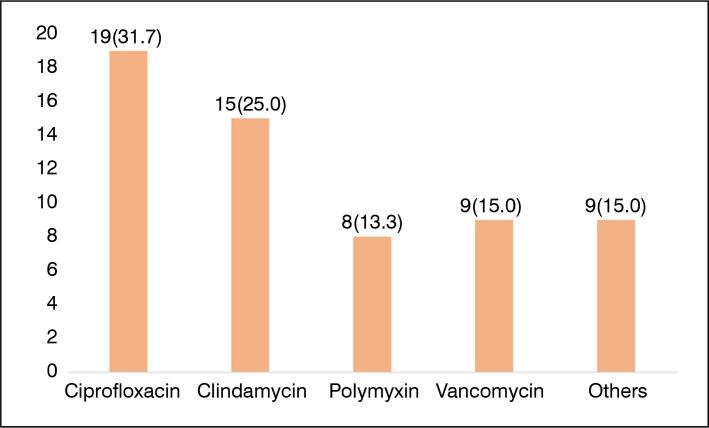
Characteristics of the antibiotics used in surgical patients resulting from hip arthroplasties, based on information from medical records of patients who underwent hip arthroplasty at Getúlio Vargas Hospital – PI, from 2020 to 2023. (n = 22).

Figure 3 shows the antibiotics used to treat infections. It is observed that Ciprofloxacin (31.7%) was the most used, followed by Clindamycin (25.0%) and Vancomycin (15.0%). The choice of antimicrobials reflects the need for coverage against Gram-negative and Gram-positive pathogens, with a focus on the main identified pathogens.

**Figure 4:**
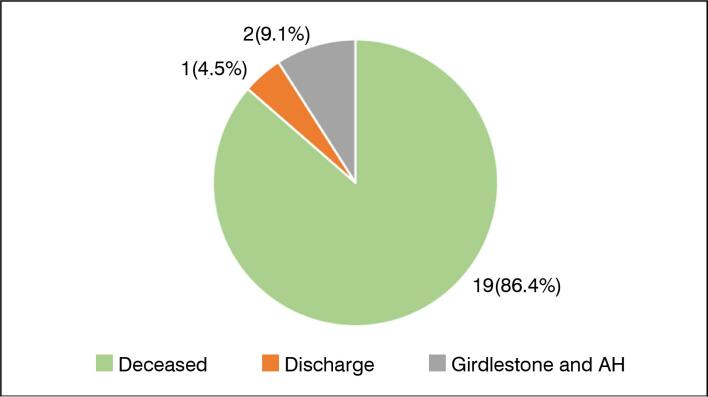
Characteristics of the outcomes of surgical site infections resulting from hip arthroplasties, based on information from medical records of patients who underwent hip arthroplasty at Getúlio Vargas Hospital – PI, from 2020 to 2023. (n = 22).

Figure 4 illustrates the outcomes of the infections. The high percentage of patients discharged (86.4%) indicates a good treatment response. However, the need for Girdlestone in two patients and the death of one patient highlight the severity of complications associated with infections.

Table 2 presents the associations between infection outcomes and clinical and sociodemographic variables.

**Table 2:** Association between the outcomes of surgical site infections resulting from hip arthroplasties, based on information from medical records of patients who underwent hip arthroplasty at Getúlio Vargas Hospital – PI, from 2020 to 2023. (n = 22).

Variables	Outcome			P-value
	High	Death	Performed Gilderstone and AH	
	N(%)	N(%)	N(%)	
**Sex**				**0,680**
Male	8(42.1)	0(0.0)	1(50.0)	
Female	11(57.9)	1(100.0)	1(50.0)	
**Age Range**				**0.600**
40-59 years	5(26.3)	0(0.0)	0(0.0)	
≥60 years	14(73.7)	1(100.0)	2(100.0)	
**Cause**				**0.270**
Hip osteoarthritis	3(15.8)	1(100.0)	0(0.0)	
Avascular necrosis	1(5.3)	0(0.0)	0(0.0)	
Trauma	15(78.9)	0(0.0)	2(100.0)	
**Anesthesia**				**-**
Spinal	19(100.0)	1(100.0)	2(100.0)	
**ASA**				**0.193**
1	4(21.1)	0(0.0)	0(0.0)	
2	10(52.6)	0(0.0)	0(0.0)	
3	5(26.3)	1(100.0)	2(100.0)	
**UTI**				**0.235**
No	10(52.6)	0(0.0)	0(0.0)	
Yes	9(47.4)	1(100.0)	2(100.0)	
**Prophylactic Antibiotic**				**0.278**
Cephalothin 48h	2(10.5)	0(0.0)	1(50.0)	
Cephalothin 24h	17(89.5)	1(100.0)	1(50.0)	
**Previous surgery**				**0.841**
No	17(89.5)	1(100.0)	2(100.0)	
Yes	2(10.5)	0(0.0)	0(0.0)	
**Previous surgery**				**-**
Dislocation revision	1(50.0)	0(0.0)	0(0.0)	
Transtrochanteric	1(50.0)	0(0.0)	0(0.0)	
**Partial or total**				**0.081**
Partial	5(26.3)	0(0.0)	2(100.0)	
Total	14(73.7)	1(100.0)	0(0.0)	
**Cement**				**0.156**
With cement	7(36.8)	0(0.0)	2(100.0)	
Without cement	12(63.2)	1(100.0)	0(0.0)	

Source: Authors (2025). ^1^Chi-square test, with Yates’ correction, at the 5% level.

It is noted that no statistically significant associations were observed between the outcomes and the analyzed variables (p > 0.05 in all comparisons). However, it was observed that death occurred in a patient with ASA 3, over 60 years of age, and a diagnosis of hip osteoarthritis, factors that may indicate a higher risk of postoperative complications.

## DISCUSSION

This study reveals relevant data on surgical site infections (SSI) and Periprosthetic Joint Infection (PJI) in patients undergoing hip arthroplasty at Getúlio Vargas Hospital from 2020 to 2023. PJI represents one of the most serious complications following hip arthroplasty, bringing significant impacts both for patients and the healthcare system due to high morbidity, mortality risk, and treatment costs. However, diagnosing this condition remains a major challenge in clinical practice, as there is no universal consensus on its definition^12^.

In this research, the observed infection rate was 2.0%, consistent with the literature, which indicates a variable incidence between 1% and 2% in large hospitals^13–16^. Despite this, it is emphasized that this data, although within the expected range, should not be underestimated, as SSIs in orthopedic procedures such as hip arthroplasty are associated with high morbidity, the need for surgical reinterventions, and, in extreme cases, death^2^.

The profile of infected patients showed a predominance of females, an average age of 68.8 years, and a higher frequency of elderly individuals (≥ 60 years), which corroborates findings from other previously conducted studies^11,15,17^. These findings indicate advanced age as a risk factor for postoperative complications due to the presence of comorbidities and lower functional reserve^18^. Additionally, most patients had an ASA classification of 2 or 3, reinforcing the previous clinical picture of systemic compromise.

It is noteworthy that the most frequent indication for performing arthroplasty was trauma, followed by hip osteoarthritis, a pattern also reported in similar studies in Brazilian public hospital contexts^19,20^. The predominance of traumatic cases may reflect the emergency nature of the service and the high demand for urgent surgeries in elderly populations, often victims of falls^21,22^.

From a technical standpoint, standardized adoption of spinal anesthesia and antibiotic prophylaxis with cephalothin for 24 hours was observed in most cases. Although this practice aligns with established protocols^22^, the occurrence of infections suggests the need for periodic reassessment of the effectiveness of the prophylactic regimen, also considering local microbiological profiles^11^.

The most frequently isolated bacteria were *Escherichia coli* and *Acinetobacter baumannii*, both known for their resistance and ability to colonize in hospital environments. The presence of *E. coli*, a typically enteric pathogen, raises the hypothesis of endogenous contamination or failures in aseptic measures in patients with immunosenescence^23^. Meanwhile, *A. baumannii* is strongly associated with difficult-to-manage nosocomial infections, requiring strict protocols for hospital infection control^24^.

It is noteworthy that PJIs caused by *A. baumannii* have garnered increasing attention in clinical practice, primarily due to its remarkable ability to develop resistance to various antibiotics and form biofilms, which significantly complicates treatment and contributes to infection persistence^24^.

Regarding treatment, the predominant use of Ciprofloxacin, Clindamycin, and Vancomycin highlights the need for broad initial empirical coverage that addresses both Gram-negative and Gram-positive bacteria. The choice of these antimicrobials seems to align with the sensitivity patterns observed in the identified pathogens, which may have contributed to the good therapeutic outcomes in most cases^25^.

Although there are international diagnostic criteria for periprosthetic infection, such as those established in 2013 by the International Consensus, which consider clinical, laboratory, and microbiological findings, their application in this study was hindered by the lack of clinical data in public databases. This limitation reinforces the need to improve and standardize national records, such as those from DATASUS, to enable analyses more aligned with international guidelines^8^.

Regarding the duration of antimicrobial therapy, a period of 4 to 12 weeks of antibiotic treatment directed against the isolated pathogens is recommended, especially after prosthesis removal in revision procedures 11. Recent evidence suggests that a six-week antibiotic regimen may be effective in this context, showing good clinical results^23,11^. However, there remains a lack of consistent data on the efficacy of antibiotic treatment in single-stage revision cases, which limits the development of standardized care in these cases. Indeed, the clinical outcome was satisfactory in most patients, with 86.4% being discharged from the hospital. However, it is important to highlight the occurrence of serious complications, such as the need for Girdlestone surgery in two patients and the death of one patient. The latter case occurred in an elderly patient, classified as ASA 3, with a diagnosis of hip osteoarthritis, suggesting that such factors may be associated with a worse prognosis, although they did not reach statistical significance in this study.

According to Shah and Parker^26^, Girdlestone resection is considered a salvage measure, usually indicated in situations where the patient has significant comorbidities that limit the performance of more complex reconstructive procedures, or when previous therapeutic approaches have failed. This technique, although associated with loss of joint function and reduced mobility, may be essential for controlling infection and improving quality of life in severe and refractory cases.

Furthermore, the absence of statistically significant associations between clinical/sociodemographic variables and outcomes may be due to the small sample size, which limits statistical power.

These findings reinforce the need for ongoing prevention strategies, including active microbiological surveillance, review of antibiotic protocols, and strengthening infection control practices, especially among vulnerable populations such as the elderly and those with comorbidities.

## CONCLUSION

The analysis of surgical site infection cases in patients undergoing hip arthroplasty at Hospital Getúlio Vargas, between 2020 and 2023, revealed an infection rate of 2.0%, a figure consistent with the literature, but still underscores the importance of preventive strategies in the surgical environment. The profile of affected patients indicated greater vulnerability among the elderly, especially females with pre-existing clinical comorbidities, reflected in ASA classifications 2 and 3.

From a clinical perspective, the predominance of surgeries performed due to trauma and the high need for admission to intensive care units reinforce the severity of the cases. The presence of multidrug-resistant microorganisms, such as *Acinetobacter baumannii* and *Escherichia coli*, highlights the challenge in therapeutic management, requiring antimicrobial regimens with broad coverage. Despite this, most patients showed favorable progression, with hospital discharge in 86.4% of cases, although there were significant complications, such as the need for prosthesis resection (Girdlestone) and one case of death.

The absence of statistically significant associations between outcomes and the analyzed variables may be related to the small number of cases with infection, but it does not exclude the clinical relevance of factors such as advanced age, high ASA status, and previous joint diseases as potential aggravating factors.

In light of this, the findings reinforce the need for continuous surveillance, infection prevention and control protocols, as well as a multidisciplinary approach in perioperative care, especially for patients with higher clinical risk. Studies with larger sample sizes and longitudinal follow-up may contribute to a deeper understanding of factors associated with joint prosthesis infection and improve surgical outcomes.

## Data Availability

The underlying contents of the research text are contained in the manuscript.
